# Supplementary material for: Pyruvate Dehydrogenase Kinase 1 inhibition mediated oxidative phosphorylation enhancement in cartilage promotes osteoarthritis progression
Source: BMC Musculoskelet Disord. 2023 Jul 20;24:597. doi: 10.1186/s12891-023-06585-6 (PMC10357736; doi:10.1186/s12891-023-06585-6)
Supplement: Supplementary file 1 — Additional file 1. [file 12891_2023_6585_MOESM1_ESM.zip › BP.Gsea.1653623667859/GOBP_MITOCHONDRIAL_TRANSLATION.html]

Details for gene set GOBP\_MITOCHONDRIAL\_TRANSLATION[GSEA]

|  || Dataset | 55457\_collapsed\_to\_symbols.cls.cls #OA\_versus\_normal.cls.cls #OA\_versus\_normal\_repos |
| Phenotype | cls.cls#OA\_versus\_normal\_repos |
| Upregulated in class | OA |
| GeneSet | GOBP\_MITOCHONDRIAL\_TRANSLATION |
| Enrichment Score (ES) | 0.525611 |
| Normalized Enrichment Score (NES) | 2.1192002 |
| Nominal p-value | 0.0 |
| FDR q-value | 0.005651081 |
| FWER p-Value | 0.098 |
Table: GSEA Results Summary

  

Fig 1: Enrichment plot: GOBP\_MITOCHONDRIAL\_TRANSLATION      
 Profile of the Running ES Score & Positions of GeneSet Members on the Rank Ordered List

  

| SYMBOL | TITLE | RANK IN GENE LIST | RANK METRIC SCORE | RUNNING ES | CORE ENRICHMENT || 1 | MRPL16 | mitochondrial ribosomal protein L16 [Source:HGNC Symbol;Acc:HGNC:14476] | 132 | 0.874 | 0.0435 | Yes |
| 2 | TUFM | "Tu translation elongation factor, mitochondrial [Source:HGNC Symbol;Acc:HGNC:12420]" | 189 | 0.828 | 0.0901 | Yes |
| 3 | MRPS14 | mitochondrial ribosomal protein S14 [Source:HGNC Symbol;Acc:HGNC:14049] | 207 | 0.814 | 0.1388 | Yes |
| 4 | MRPL57 | mitochondrial ribosomal protein L57 [Source:HGNC Symbol;Acc:HGNC:14514] | 314 | 0.740 | 0.1760 | Yes |
| 5 | COA3 | cytochrome c oxidase assembly factor 3 [Source:HGNC Symbol;Acc:HGNC:24990] | 597 | 0.625 | 0.1925 | Yes |
| 6 | NGRN | "neugrin, neurite outgrowth associated [Source:HGNC Symbol;Acc:HGNC:18077]" | 741 | 0.583 | 0.2173 | Yes |
| 7 | TACO1 | translational activator of cytochrome c oxidase I [Source:HGNC Symbol;Acc:HGNC:24316] | 923 | 0.536 | 0.2361 | Yes |
| 8 | MRPS16 | mitochondrial ribosomal protein S16 [Source:HGNC Symbol;Acc:HGNC:14048] | 994 | 0.519 | 0.2626 | Yes |
| 9 | MTIF2 | mitochondrial translational initiation factor 2 [Source:HGNC Symbol;Acc:HGNC:7441] | 1043 | 0.508 | 0.2902 | Yes |
| 10 | NDUFA7 | NADH:ubiquinone oxidoreductase subunit A7 [Source:HGNC Symbol;Acc:HGNC:7691] | 1097 | 0.496 | 0.3166 | Yes |
| 11 | TSFM | "Ts translation elongation factor, mitochondrial [Source:HGNC Symbol;Acc:HGNC:12367]" | 1120 | 0.492 | 0.3451 | Yes |
| 12 | QRSL1 | glutaminyl-tRNA amidotransferase subunit QRSL1 [Source:HGNC Symbol;Acc:HGNC:21020] | 1250 | 0.467 | 0.3638 | Yes |
| 13 | MRPL58 | mitochondrial ribosomal protein L58 [Source:HGNC Symbol;Acc:HGNC:5359] | 1298 | 0.459 | 0.3884 | Yes |
| 14 | MRPS11 | mitochondrial ribosomal protein S11 [Source:HGNC Symbol;Acc:HGNC:14050] | 1305 | 0.457 | 0.4160 | Yes |
| 15 | SHMT2 | serine hydroxymethyltransferase 2 [Source:HGNC Symbol;Acc:HGNC:10852] | 1518 | 0.426 | 0.4257 | Yes |
| 16 | RCC1L | RCC1 like [Source:HGNC Symbol;Acc:HGNC:14948] | 1559 | 0.419 | 0.4484 | Yes |
| 17 | MRPS7 | mitochondrial ribosomal protein S7 [Source:HGNC Symbol;Acc:HGNC:14499] | 1650 | 0.406 | 0.4664 | Yes |
| 18 | IARS2 | "isoleucyl-tRNA synthetase 2, mitochondrial [Source:HGNC Symbol;Acc:HGNC:29685]" | 1685 | 0.401 | 0.4884 | Yes |
| 19 | MRPS28 | mitochondrial ribosomal protein S28 [Source:HGNC Symbol;Acc:HGNC:14513] | 1865 | 0.377 | 0.4977 | Yes |
| 20 | MRPL23 | mitochondrial ribosomal protein L23 [Source:HGNC Symbol;Acc:HGNC:10322] | 2210 | 0.337 | 0.4916 | Yes |
| 21 | MRPS18A | mitochondrial ribosomal protein S18A [Source:HGNC Symbol;Acc:HGNC:14515] | 2295 | 0.327 | 0.5052 | Yes |
| 22 | FASTKD3 | FAST kinase domains 3 [Source:HGNC Symbol;Acc:HGNC:28758] | 2473 | 0.306 | 0.5102 | Yes |
| 23 | GATB | glutamyl-tRNA amidotransferase subunit B [Source:HGNC Symbol;Acc:HGNC:8849] | 2515 | 0.302 | 0.5256 | Yes |
| 24 | WARS2 | "tryptophanyl tRNA synthetase 2, mitochondrial [Source:HGNC Symbol;Acc:HGNC:12730]" | 3014 | 0.248 | 0.5021 | No |
| 25 | C1QBP | complement C1q binding protein [Source:HGNC Symbol;Acc:HGNC:1243] | 3083 | 0.241 | 0.5117 | No |
| 26 | MRPL44 | mitochondrial ribosomal protein L44 [Source:HGNC Symbol;Acc:HGNC:16650] | 3306 | 0.220 | 0.5080 | No |
| 27 | SARS2 | "seryl-tRNA synthetase 2, mitochondrial [Source:HGNC Symbol;Acc:HGNC:17697]" | 3411 | 0.210 | 0.5128 | No |
| 28 | MRPS15 | mitochondrial ribosomal protein S15 [Source:HGNC Symbol;Acc:HGNC:14504] | 3475 | 0.205 | 0.5205 | No |
| 29 | NSUN3 | NOP2/Sun RNA methyltransferase 3 [Source:HGNC Symbol;Acc:HGNC:26208] | 3612 | 0.192 | 0.5217 | No |
| 30 | GFM1 | G elongation factor mitochondrial 1 [Source:HGNC Symbol;Acc:HGNC:13780] | 4011 | 0.155 | 0.5003 | No |
| 31 | MRPS27 | mitochondrial ribosomal protein S27 [Source:HGNC Symbol;Acc:HGNC:14512] | 4088 | 0.147 | 0.5035 | No |
| 32 | DARS2 | "aspartyl-tRNA synthetase 2, mitochondrial [Source:HGNC Symbol;Acc:HGNC:25538]" | 4480 | 0.115 | 0.4801 | No |
| 33 | ALKBH1 | "alkB homolog 1, histone H2A dioxygenase [Source:HGNC Symbol;Acc:HGNC:17911]" | 4765 | 0.090 | 0.4635 | No |
| 34 | MRPS17 | mitochondrial ribosomal protein S17 [Source:HGNC Symbol;Acc:HGNC:14047] | 4898 | 0.080 | 0.4581 | No |
| 35 | RMND1 | required for meiotic nuclear division 1 homolog [Source:HGNC Symbol;Acc:HGNC:21176] | 5084 | 0.066 | 0.4478 | No |
| 36 | HARS1 | histidyl-tRNA synthetase 1 [Source:HGNC Symbol;Acc:HGNC:4816] | 5196 | 0.057 | 0.4426 | No |
| 37 | TARS2 | "threonyl-tRNA synthetase 2, mitochondrial [Source:HGNC Symbol;Acc:HGNC:30740]" | 5576 | 0.026 | 0.4147 | No |
| 38 | MTG1 | mitochondrial ribosome associated GTPase 1 [Source:HGNC Symbol;Acc:HGNC:32159] | 5588 | 0.025 | 0.4154 | No |
| 39 | LARS2 | "leucyl-tRNA synthetase 2, mitochondrial [Source:HGNC Symbol;Acc:HGNC:17095]" | 7017 | -0.081 | 0.3093 | No |
| 40 | GARS1 | glycyl-tRNA synthetase 1 [Source:HGNC Symbol;Acc:HGNC:4162] | 7069 | -0.085 | 0.3106 | No |
| 41 | MRPS12 | mitochondrial ribosomal protein S12 [Source:HGNC Symbol;Acc:HGNC:10380] | 7328 | -0.104 | 0.2969 | No |
| 42 | FASTKD2 | FAST kinase domains 2 [Source:HGNC Symbol;Acc:HGNC:29160] | 7730 | -0.136 | 0.2741 | No |
| 43 | GATC | glutamyl-tRNA amidotransferase subunit C [Source:HGNC Symbol;Acc:HGNC:25068] | 7852 | -0.144 | 0.2735 | No |
| 44 | MRPS2 | mitochondrial ribosomal protein S2 [Source:HGNC Symbol;Acc:HGNC:14495] | 7863 | -0.146 | 0.2817 | No |
| 45 | MRPS18B | mitochondrial ribosomal protein S18B [Source:HGNC Symbol;Acc:HGNC:14516] | 8145 | -0.166 | 0.2701 | No |
| 46 | LRPPRC | leucine rich pentatricopeptide repeat containing [Source:HGNC Symbol;Acc:HGNC:15714] | 8249 | -0.175 | 0.2728 | No |
| 47 | PTCD3 | pentatricopeptide repeat domain 3 [Source:HGNC Symbol;Acc:HGNC:24717] | 8287 | -0.178 | 0.2808 | No |
| 48 | YARS2 | tyrosyl-tRNA synthetase 2 [Source:HGNC Symbol;Acc:HGNC:24249] | 10299 | -0.350 | 0.1459 | No |
| 49 | CDK5RAP1 | CDK5 regulatory subunit associated protein 1 [Source:HGNC Symbol;Acc:HGNC:15880] | 10870 | -0.413 | 0.1269 | No |
| 50 | MTRF1 | mitochondrial translation release factor 1 [Source:HGNC Symbol;Acc:HGNC:7469] | 11517 | -0.503 | 0.1076 | No |
Table: GSEA details [plain text format]

  

Fig 2: GOBP\_MITOCHONDRIAL\_TRANSLATION      
 Blue-Pink O' Gram in the Space of the Analyzed GeneSet

  

Fig 3: GOBP\_MITOCHONDRIAL\_TRANSLATION: Random ES distribution      
 Gene set null distribution of ES for **GOBP\_MITOCHONDRIAL\_TRANSLATION**
